# Supplementary material for: The Effect of Oral Iron Supplementation on Gut Microbial Composition: a Secondary Analysis of a Double-Blind, Randomized Controlled Trial among Cambodian Women of Reproductive Age
Source: Microbiol Spectr. 2023 May 18;11(3):e05273-22. doi: 10.1128/spectrum.05273-22 (PMC10269596; doi:10.1128/spectrum.05273-22)
Supplement: Supplemental file 1 — Supplemental material. Download spectrum.05273-22-s0001.pdf, PDF file, 0.6 MB [file spectrum.05273-22-s0001.pdf]

SUPPLEMENTAL FIGURES

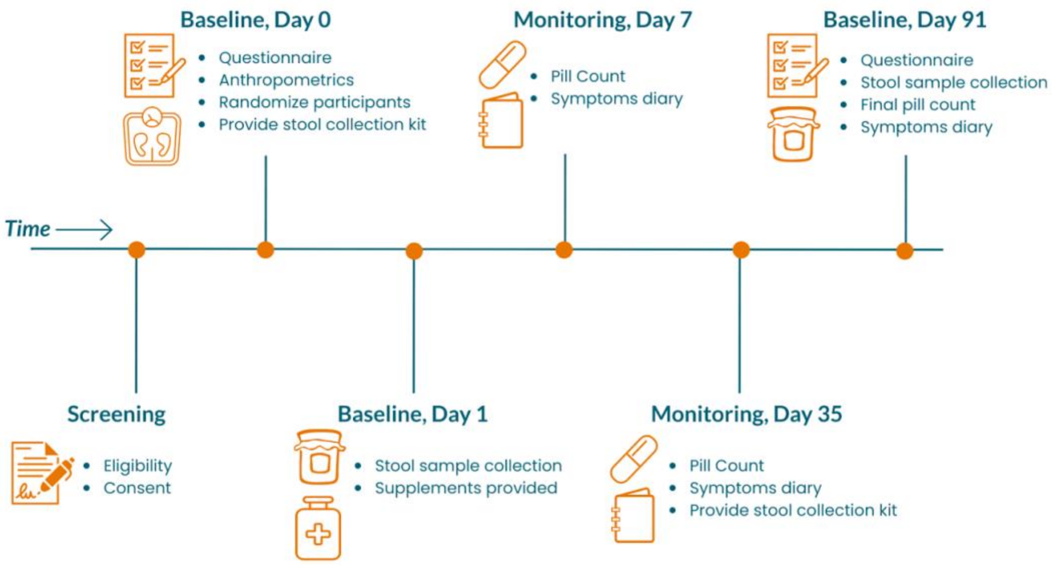

Fig. S1. Trial timeline

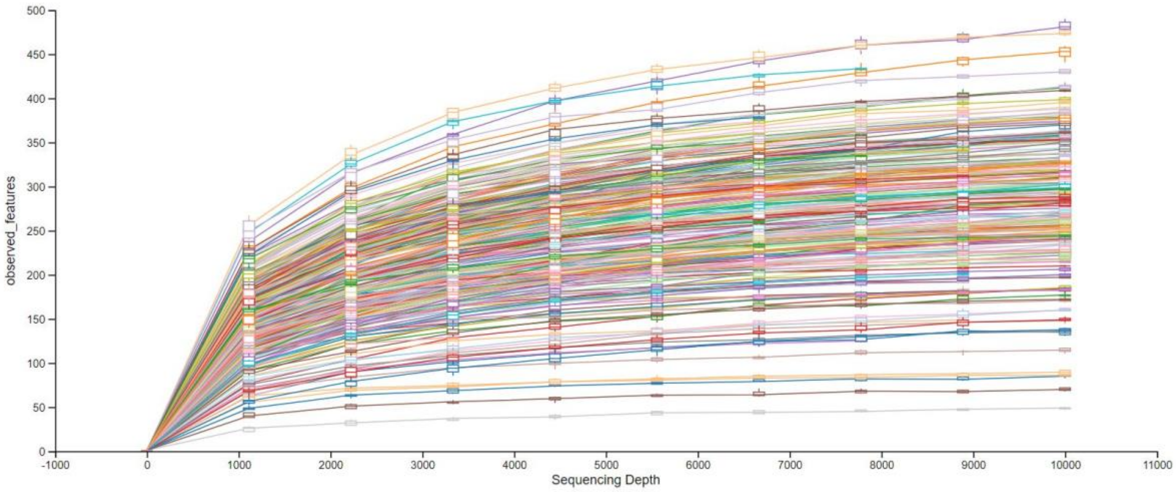

Fig. S2. Alpha rarefaction curve of the 172 stool samples sequenced by 16S rRNA sequencing. Each line represents the function of the observed number of OTUs over the sequences per samples.

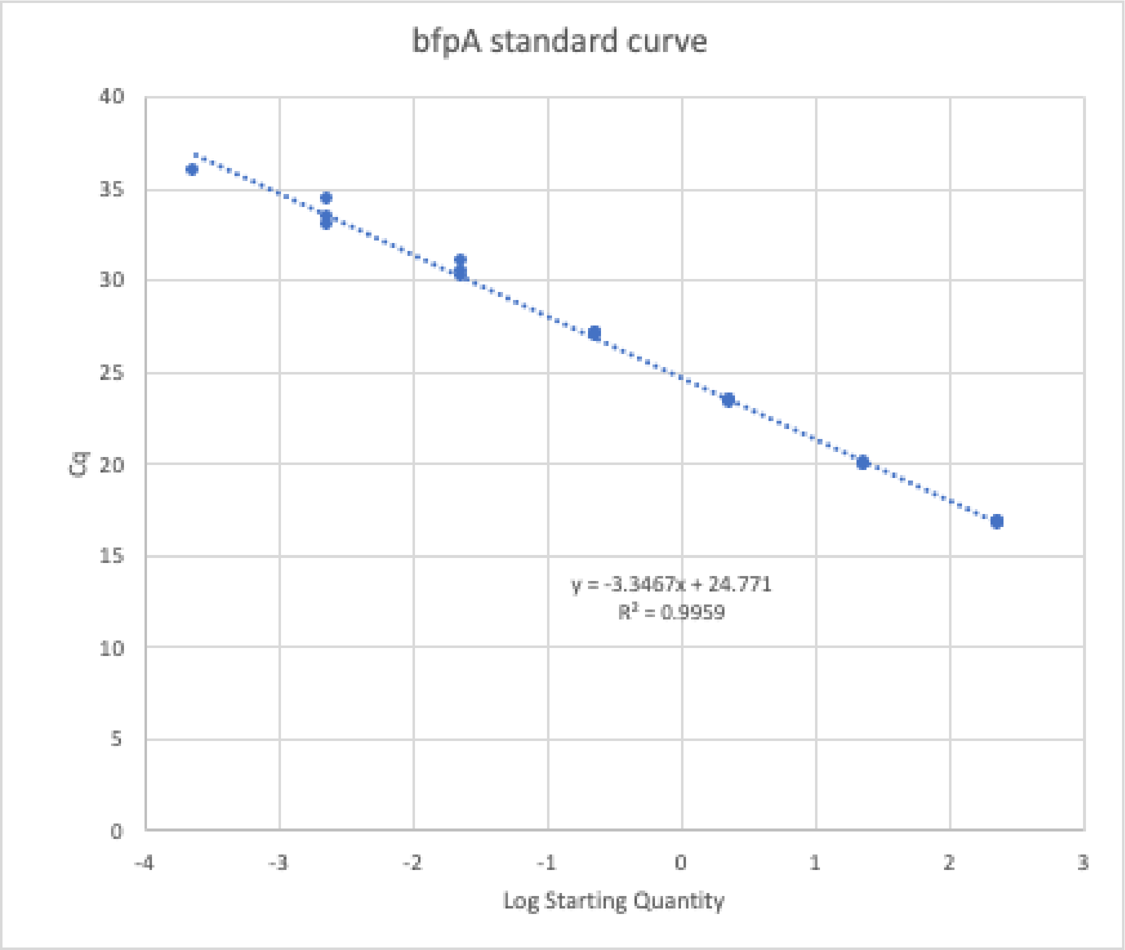

Fig. S3. *bfpa* standard curve

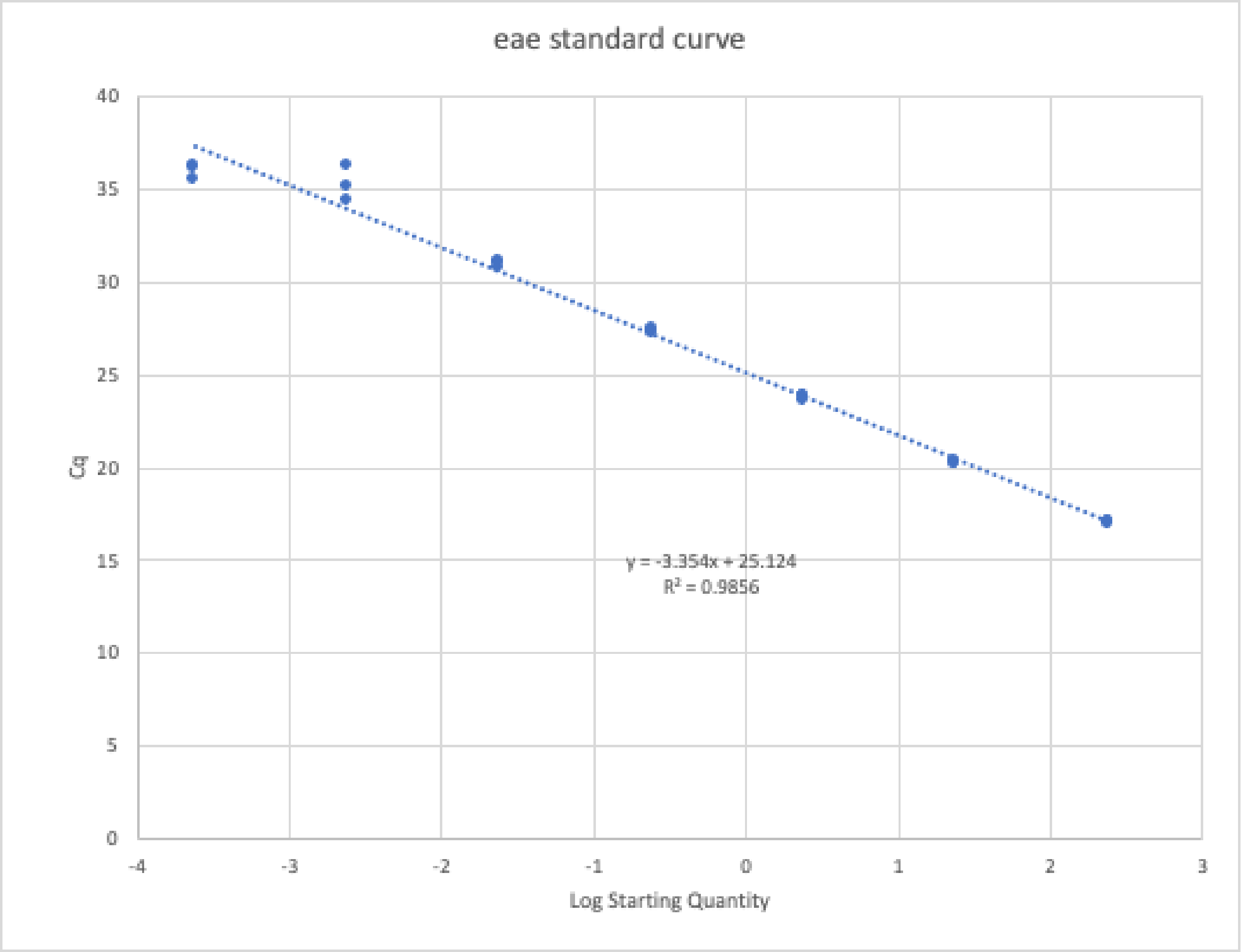

Fig. S4. *eae* standard curve

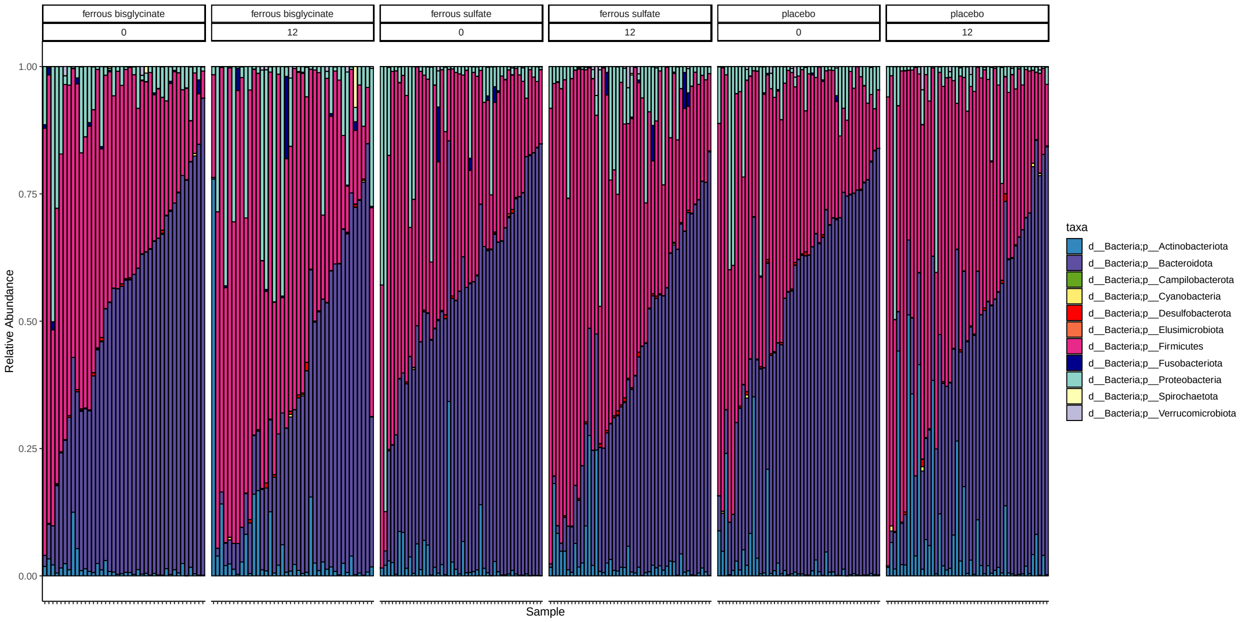

Fig. S5. Stacked bar chart showing the relative abundance of phyla in 172 samples from Cambodian WRA organized by trial arm and week.

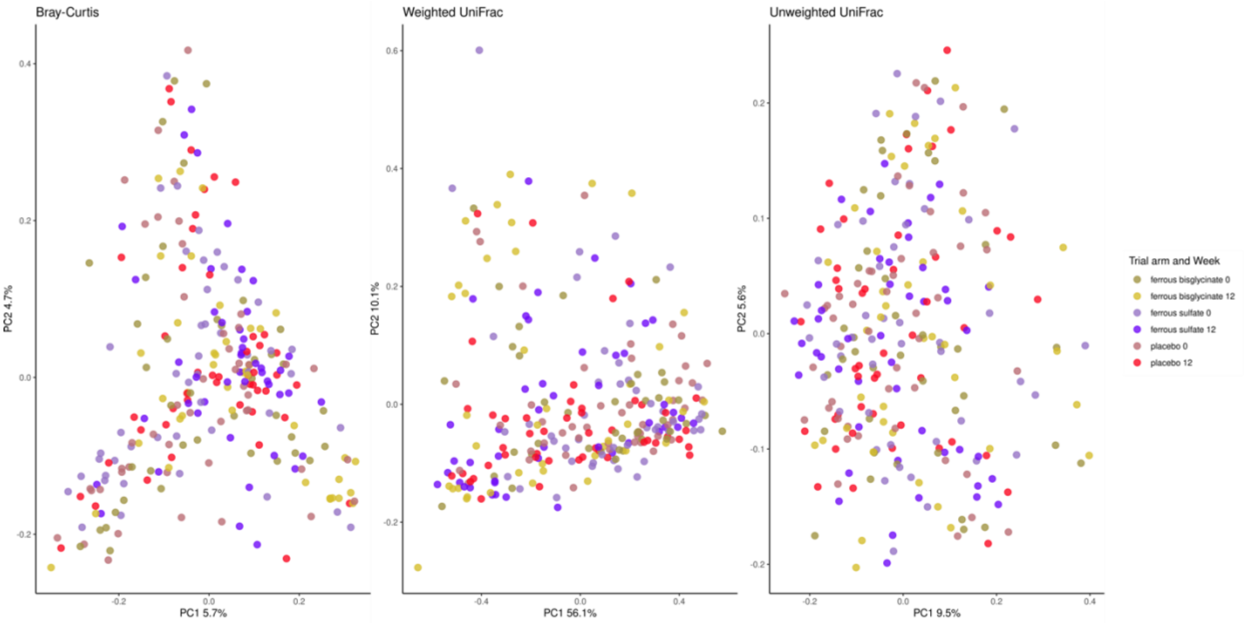

Fig. S6. Further visual examination through principal coordinate analysis plotting and PERMANOVA testing showed no significant interaction between the week and trial arm for weighted UniFrac, unweighted UniFrac, and Bray-Curtis dissimilarity.

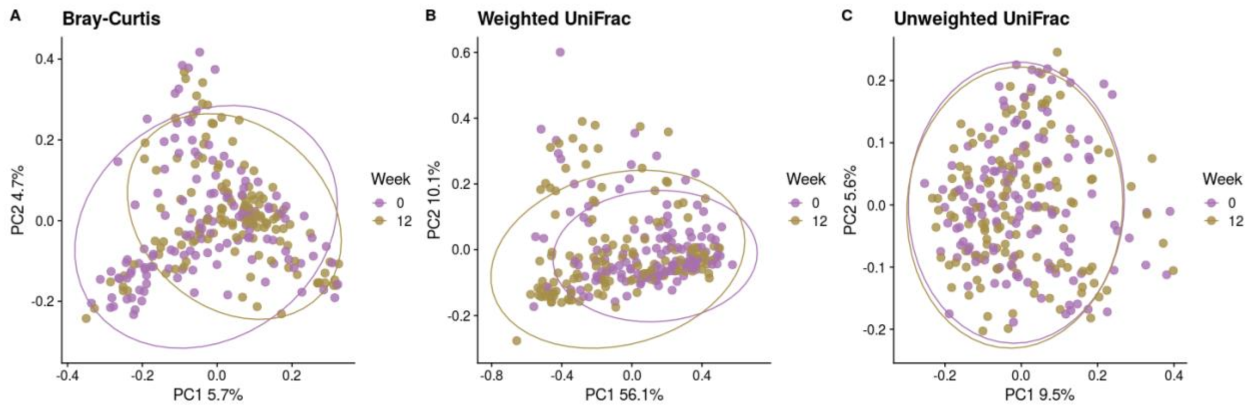

Fig. S7. Weighted beta diversity significantly changes between trial weeks regardless of arm.

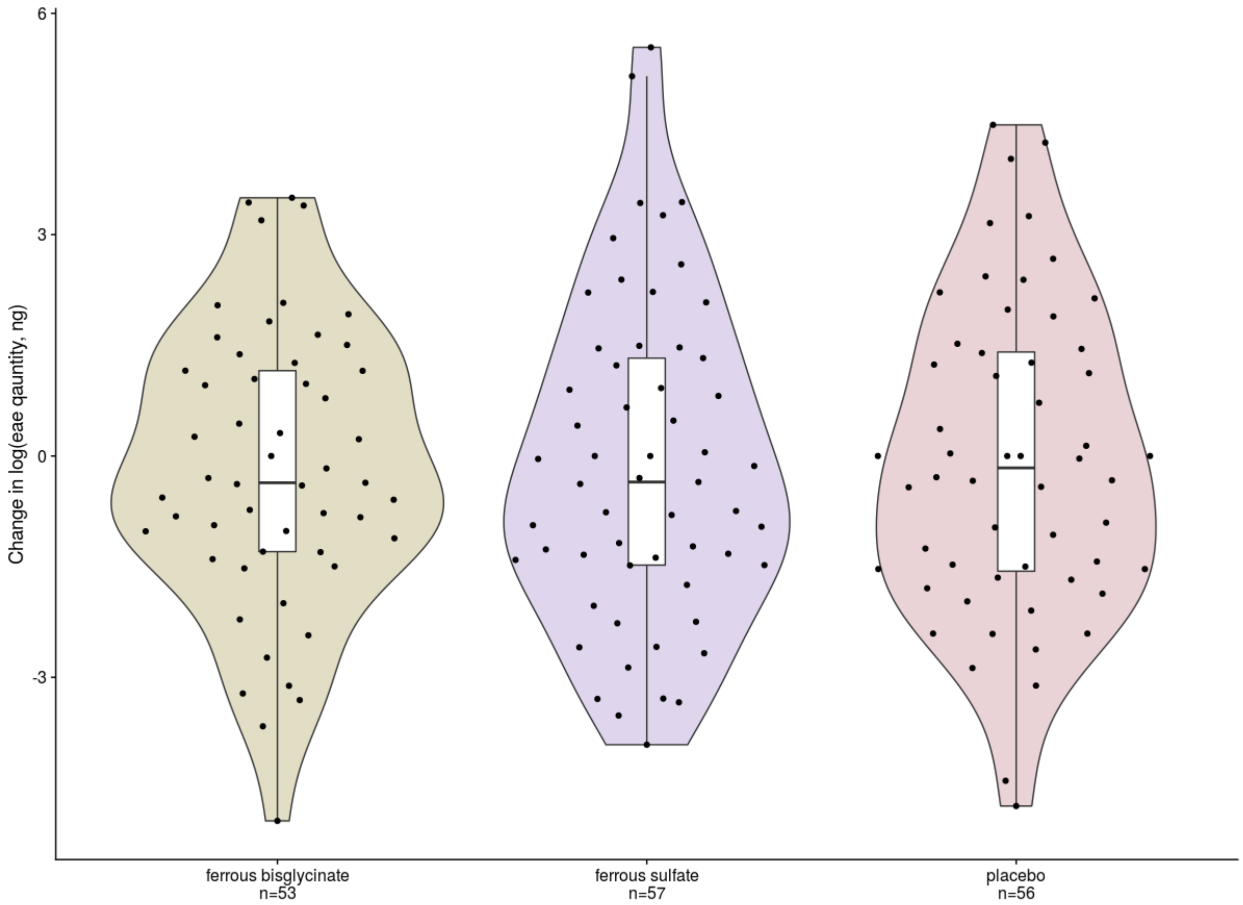

Fig. S8. Estimate change in *eae* presence over intervention.

SUPPLEMENTAL TABLES

Table S1. Mixed effects logistic regression on the presence of *bfpa* across treatment groups and time.

Term	Estimate	Standard Error	Statistic	P-Value	Confidence Low	Confidence High	Group
(Intercept)	-6.17710	1.23657	-4.99534	0.00000	-8.60074	-3.75346	fixed
Week12	-2.80875	1.36129	-2.06330	0.03908	-5.47682	-0.14067	fixed
Ferrous bisglycinate (baseline)	-0.51515	1.21513	-0.42395	0.67161	-2.89675	1.86646	fixed
Ferrous sulfate (baseline)	-3.00772	1.71192	-1.75692	0.07893	-6.36303	0.34759	fixed
Ferrous bisglycinate (12 week)	1.76339	1.67362	1.05364	0.29205	-1.51684	5.04362	fixed
Ferrous sulfate (12 week)	5.45731	2.13221	2.55946	0.01048	1.27825	9.63637	fixed
sd_(intercept). Patint_ID	6.87219	NA	NA	NA	NA	NA	Patint_ ID

Table S2. Number of qPCR samples that tested positive for both *eae* and *bpfA* (*n=166*) as recorded by trial arm and week (0 and 12).

**Arm**	**Week**	**Number of samples positive for both *eae* and *bpfA* (*n*=166)**
Placebo	0	9
	12	4
Ferrous bisglycinate	0	6
	12	4
Ferrous sulfate	0	3
	12	7
